# Pulmonary arterial hypertension due to antiphospholipid syndrome initially mimicking chronic thromboembolic pulmonary hypertension

**DOI:** 10.1186/s40885-021-00191-1

**Published:** 2022-04-01

**Authors:** Jina Yeo, Nami Shin, Kyung-Jin Ahn, Miryoung Seo, Albert Youngwoo Jang, Minsu Kim, Wook-Jin Chung

**Affiliations:** 1 Department of Internal Medicine, Gachon University Gil Medical Center, Incheon, Republic of Korea; 2 Department of Pediatrics, Division of Pediatric Cardiology, Gachon University Gil Medical Center, Incheon, Republic of Korea; 3 Gachon Cardiovascular Research Institute, Gachon University, Incheon, Republic of Korea; 4 Division of Rheumatology, Department of Internal Medicine, Gachon University Gil Medical Center, Incheon, Republic of Korea; 5 Department of Cardiovascular Medicine, Gachon University Gil Medical Center, Incheon, Republic of Korea

**Keywords:** Pulmonary arterial hypertension, Antiphospholipid syndrome, Thromboembolism, Endothelin receptor antagonist

## Abstract

Pulmonary arterial hypertension (PAH) is the second most common lung complication in antiphospholipid syndrome (APS) patients. However, the concurrent development of APS-related nonthrombotic PAH is rarely reported. Lack of awareness for group 1 PAH in APS patient may contribute to underdiagnosis of this condition. Herein, we reviewed the case of a 56-year-old female who was diagnosed with PAH related to APS that mimicked chronic thromboembolic pulmonary hypertension (CTEPH). It is crucial to be aware of the possibility of a group 1 PAH diagnosis, even though patients have already been diagnosed with CTEPH. Furthermore, a multidisciplinary approach and serial follow-up right heart catheterization with echocardiography are important to make a timely diagnosis and provide optimal treatment for APS-related PAH in patients with CTEPH-like clinical features.

## Letter to the Editor

Antiphospholipid syndrome (APS) is characterized by recurrent vascular thrombosis associated with antiphospholipid antibodies (aPLs). These aPLs induce endothelial activation and accelerate atherosclerotic arterial diseases, sustaining a proadhesive, proinflammatory, and procoagulant state [1]. Various pulmonary manifestations associated with APS have been described, including pulmonary thromboembolism (PTE), pulmonary hypertension, and acute respiratory distress syndrome [2]. Classification criteria have demonstrated that aPLs should be presented on two or more occasions that are at least 12 weeks apart [3]. Although the presence of aPLs without the relevant clinical manifestations is not enough to classify a patient as having APS, patients with APS can have aPLs-related nonthrombotic vasculopathy. Recently, aPLs have been reported to directly induce the proliferation of vascular cells in the intima and media, leading to nonthrombotic vasculopathy [4, 5]. Pulmonary arterial hypertension (PAH) can manifest in APS patients and is the second most common lung complication of patients with the syndrome, with a prevalence ranging between 1.8 and 3.5% [2]. Classical APS mainly manifests as PTE and chronic thromboembolic pulmonary hypertension (CTEPH). Patients might present PAH following pulmonary embolism associated with thrombotic APS. However, the concurrent development of APS-related nonthrombotic PAH is rarely reported. Here, we report a case of a middle-aged woman who presented with group 1 PAH related to APS that mimicked CTEPH.

**Fig. 1:**
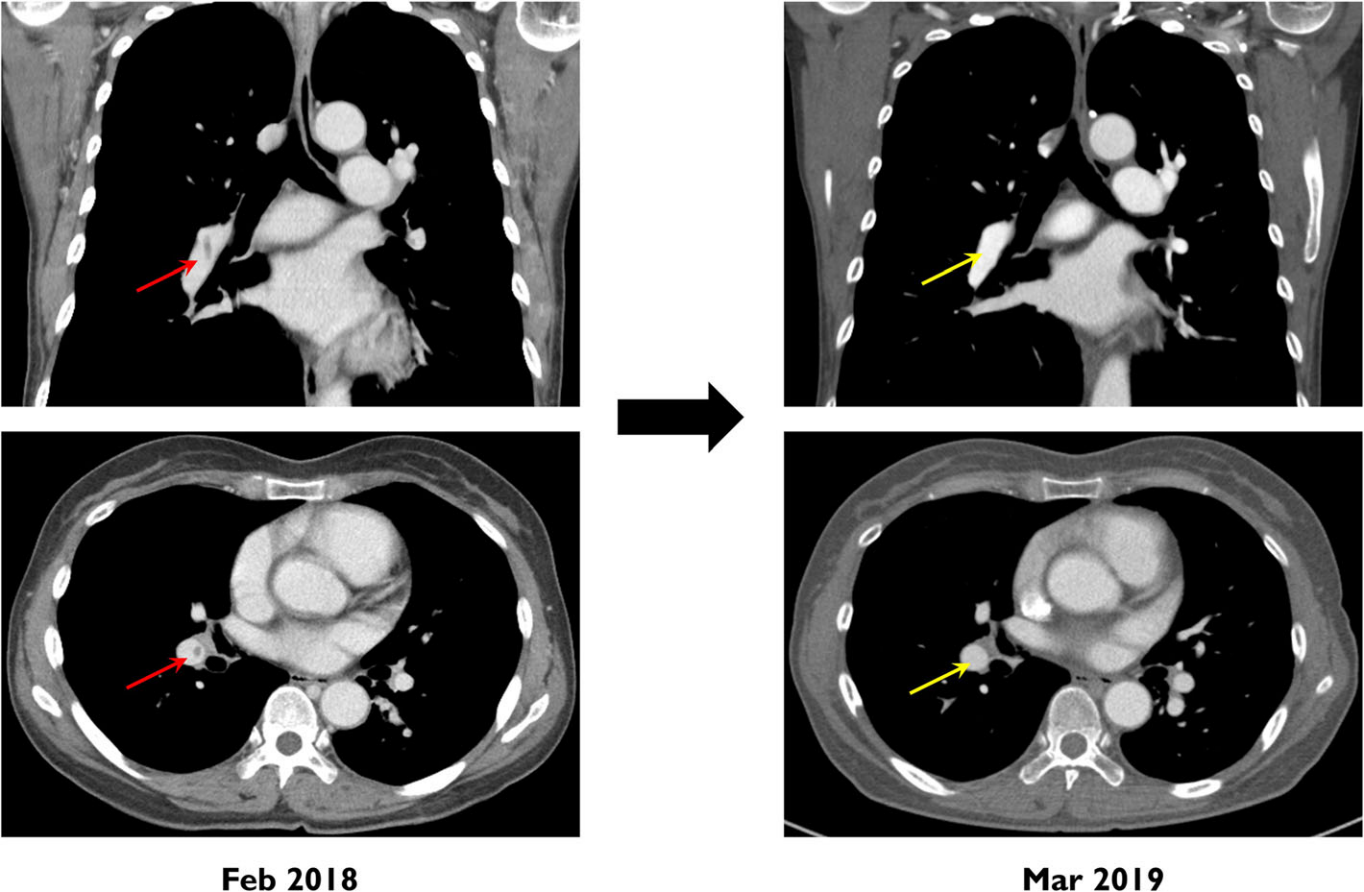
Initial and follow-up chest CT scans. The initial chest CT scan with contrast showed filling defects in right lower segmental pulmonary arteries (left panel, red arrow) and the follow-up CT scan performed 13 months after anticoagulation therapy showed complete resolution of filling defects of right lower segmental pulmonary arteries (right panel, yellow arrow)

**Fig. 2:**
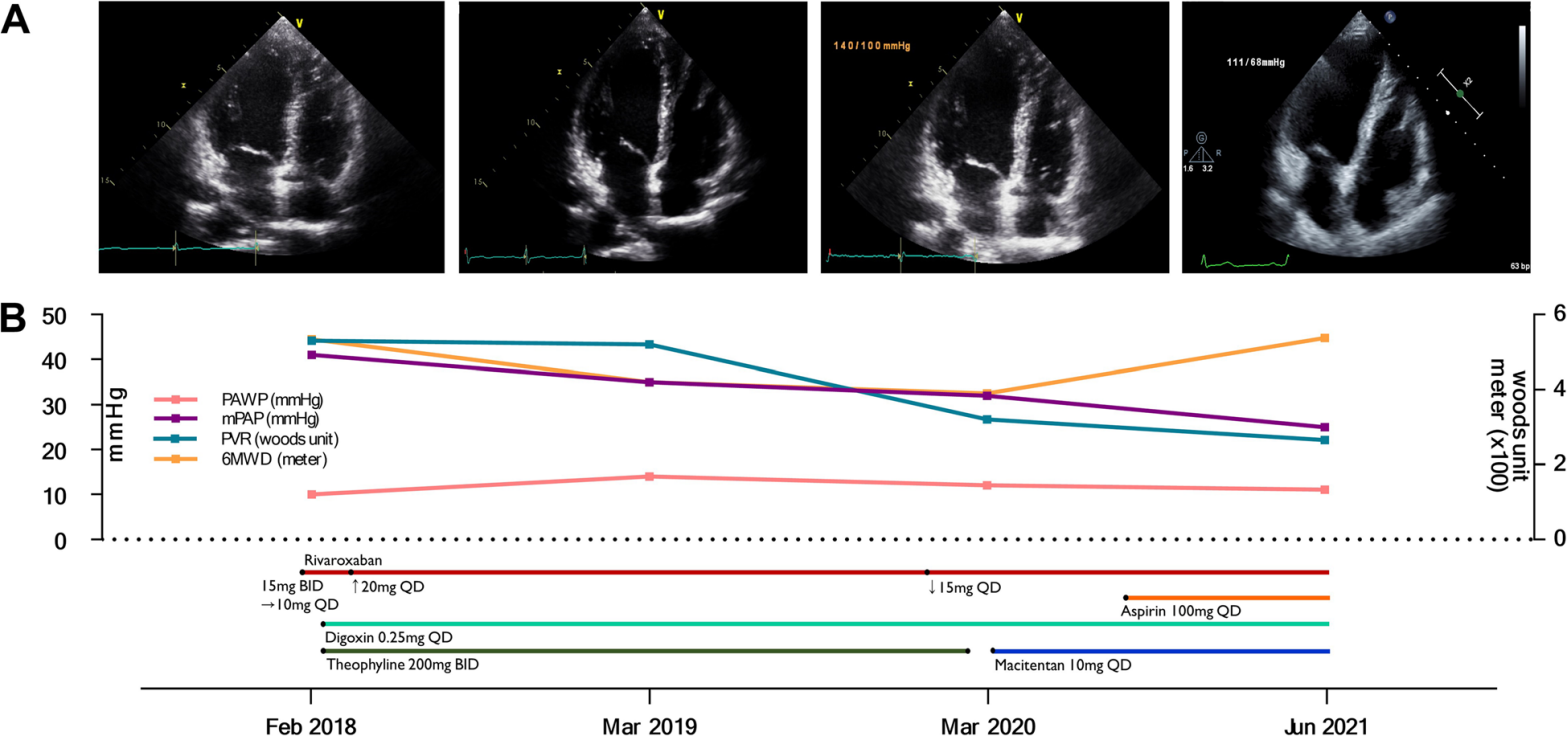
Serial changes in the echocardiography images (**A**), hemodynamic parameters of right heart catheterization and 6MWD (**B**). **A** Echocardiography images showed an enlarged right ventricle and atrium at the first three time points. After 38 months (Jun 2021), a follow-up echocardiogram showed a normal-sized right ventricle. **B** Hemodynamic parameters of right heart catheterization: PAWP, mPAP, PVR, and 6MWD showed marked improvement at the end of the follow-up period (Jun 2021). mPAP, mean pulmonary arterial pressure; PAWP, pulmonary arterial wedge pressure; PVR, pulmonary vascular resistance; and 6MWD, six-minute walking distance

A 56-year-old woman presented with progressive dyspnea on exertion for 3 months. She was able to climb no more than three flights of stairs. There was no history of fever, calf pain, surgery, prolonged immobilization, or any other comorbidity. Chest computed tomography (CT), performed at another hospital, revealed enlarged right heart chamber and filling defects in right lower segmental pulmonary arteries, suggestive of PTE (Fig. 1A, red arrows), and rivaroxaban was administered at 15 mg twice daily for 10 days. Given the development of subconjunctival hemorrhage, the patient elected to receive reduced dose of rivaroxaban (15 mg once daily). Two weeks later, she was referred to the pulmonary hypertension clinic of our hospital for further evaluation of dyspnea and unprovoked PTE. At admission, the patient was in World Health Organization functional class II. On physical examination, the blood pressure 114/80 mmHg, the respiratory rate 20 breaths/minute, and the oxygen saturation 95%. Her heart rate was regular with 86 beats/minute, but cardiac murmur was heard at tricuspid valve area. Electrocardiography, chest X-ray, and pulmonary function test were normal. Lung perfusion scan showed decreased perfusion in the right upper and lower lobes. Echocardiography demonstrated increased right ventricular systolic pressure of 51 mmHg with normal left ventricular function and right heart catheterization revealed precapillary pulmonary hypertension; the mean pulmonary arterial pressure (mPAP) was 41 mmHg, pulmonary arterial wedge pressure (PAWP) was 10 mmHg, and pulmonary vascular resistance (PVR) was 5.3 Woods unit. The six-minute walking distance (6MWD) was 534 m (Fig. 2A and B). Blood tests for hypercoagulable diseases revealed **persistent positivity for lupus anticoagulant**, while the levels of protein C, protein S, and anticardiolipin and anti-β2 glycoprotein-I antibodies were negative. Antinuclear antibody was reportedly positive, but the titer was nonspecific. Considering all of these findings, we provisionally diagnosed with CTEPH associated with primary APS. The daily dose of rivaroxaban was carefully increased to 20 mg combined with 400 mg/day of theophyline and 0.25 mg/day of digoxin. After 13 months, a chest CT showed the disappearance of filling defects in both lower segmental pulmonary arteries (Fig. 1A, yellow arrows), but right heart catheterization revealed remaining PAH; the mPAP was 35 mmHg, PAWP was 14 mmHg, and PVR was 5.2 Woods unit (Fig. 2B). After 25 months, her dyspnea was aggravated and the 6MWD was reported to be 390 m. Pulmonary hypertension was slightly improved but still remained by right heart catheterization; mPAP was 32 mmHg, PAWP was 12 mmHg, and PVR was 3.2 Woods unit (Fig. 2B). Considering all results of serial echocardiography and right heart catheterization, the final diagnosis of nonthrombotic PAH related to primary APS was made rather than CTEPH. She was started to receive PAH-specific medication, 10 mg/day of macitentan. Meanwhile, she elected to continue rivaroxaban with concomitant low dose of aspirin for secondary prevention of thromboembolism. After 40 months, a follow-up echocardiography and right heart catheterization showed a normal-sized right ventricle and marked improvement in PAH (the mPAP was 25 mmHg, PAWP was 11 mmHg, and PVR was 2.5 Woods unit; Fig. 2A and B).

This case shows PTE from PAH associated with primary APS that initially mimicked CTEPH. The mortality of PAH remains high, but aPLs-PAH related mortality is unclear [6, 7]. Lack of awareness for group 1 PAH in APS patient may contribute to underdiagnosis of this condition. Clinicians should be aware of the possibility of a group 1 PAH diagnosis, even though some patients have already been diagnosed with CTEPH, especially in patients who showed insufficient response to standard anticoagulation therapy [8]. While previous study reported that anticoagulation improves mortality rates in patients with APS and group 1 PAH, most patients need the PAH specific therapies [9]. Unfortunately, as the treatment of aPLs-PAH are based on relatively weak evidence, further studies are needed for specific treatment of PAH in patients with aPLs-PAH. A multidisciplinary approach and serial follow-up with echocardiography and right heart catheterization should be considered in these patients to make a timely diagnosis and provide optimal treatment for patients with PAH associated with primary APS that mimics CTEPH.

## Data Availability

Not applicable.

## Abbreviations

aPLs: Antiphospholipid antibodies
APS: Antiphospholipid syndrome
CT: Computed tomography
CTEPH: Chronic thromboembolic pulmonary hypertension
mPAP: Mean pulmonary arterial pressure
PAH: Pulmonary arterial hypertension
PAWP: Pulmonary arterial wedge pressure
PTE: Pulmonary thromboembolism
PVR: Pulmonary vascular resistance
6MWD: Six-minute walking distance
